# Supplementary material for: RCB initiates Arabidopsis thermomorphogenesis by stabilizing the thermoregulator PIF4 in the daytime
Source: Nat Commun. 2021 Apr 6;12:2042. doi: 10.1038/s41467-021-22313-x (PMC8024306; doi:10.1038/s41467-021-22313-x)
Supplement: Supplementary file 1 — Supplementary Information [file 41467_2021_22313_MOESM1_ESM.pdf]

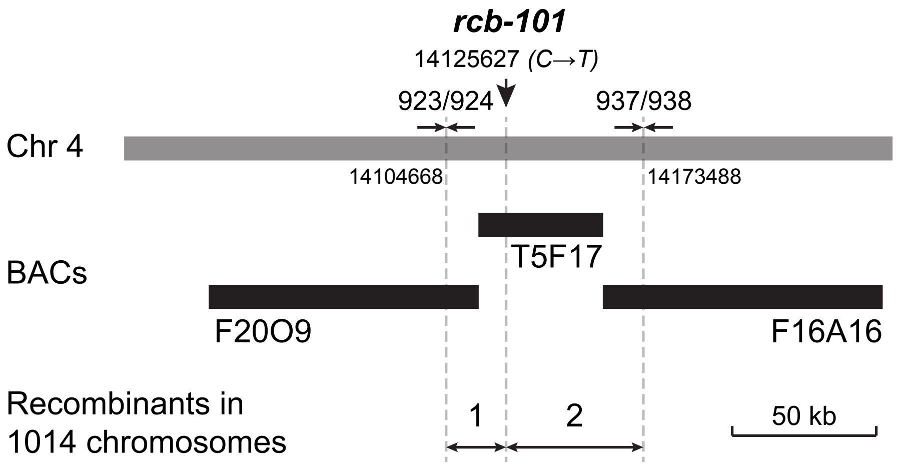

Supplementary Fig. 1. Map-based cloning of *rcb-101*. The *rcb-101* mutation was mapped to a 70-kb region on chromosome 4 between polymorphic markers 923/924 and 937/938 (Supplementary Table 1). The F2 mapping population was generated by crossing *rcb-101/hmr-22* (Col-0) and *hmr-1* (L*er*). Three recombinants out of 1014 chromosomes were found within the 70 kb region: one between marker 923/924 and the *rcb-101* mutation and two between the *rcb-101* mutation and marker 937/938.

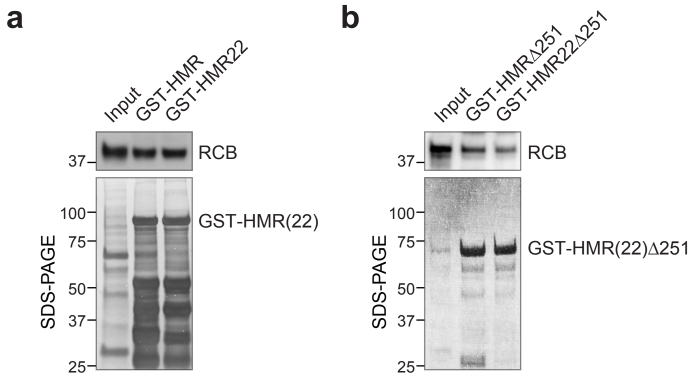

Supplementary Fig. 2. HMR22 does not significantly alter the HMR-RCB interaction. a, b GST pulldown assays using GST-tagged full-length (**a**) or Δ251 (**b**) of HMR or HMR22 to pull down in vitro translated HA-tagged full-length RCB. The upper panels are immunoblots using anti-RCB antibodies showing the bound and input fractions of HA-tagged full-length RCB; the lower panels are Coomassie Blue-stained SDS-PAGE gels showing immobilized GST-tagged HMR and HMRΔ251. The immunoblot experiments were independently repeated twice, and the results of one representative experiment are shown.

Supplementary Table 1. Markers used for map-based cloning.

Marker	Primer Names	Primers	Polymorphism between Col-0 and L*er*
923/924	923	CTATCACGTTATTTCATGCGTTC	Col-0: 147 bp
	924	GTGGACCCAGTGCTAAAGCAC	L*er*: 135 bp
937/938	937	CTGGAGTGGATGATATGACAAG	Col-0: no cut (EcoRV), 237 bp
	938	GTATAAGTCTGGAAGAATAG	Ler: cut (EcoRV), 160 bp and 77 bp

Supplementary Table 2. Primers used in qRT-PCR analyses.

Accession	Gene name	Forward primer	Reverse primer
AT1G69960	*PP2A*	TATCGGATGACGATTCTTCGTGCAG	GCTTGGTCGACTATCGAATGAGAG
AT2G43010	*PIF4*	AACCAGATCATCTCCGACCGGTTT	TCCCGCCGGTGAACTAAATCTCAA
AT4G28720	*YUC8*	TGAAACAAAACAACCCACGA	TTGATTCGCTTTGGGTCTTC
AT3G15540	*IAA19*	ATCGGTGTGGCCTTGAAAG	AACATCCCCCAAGGTACATC
AT4G32280	*IAA29*	CACCATCATTGCCCGTATCA	CCACAGTAGCCGTTGTTGGA
AT5G64120	*PRX71*	GTGACACAGTCATTCTCACT	TTGTTGAACGGCAACGGAGT
AT1G03790	*SOMNUS*	AGCAATCAGCGTCTCCATCTCCAG	TCAAGTCAAGAGATCATTGACCCATCC
AT1G28330	*DRM1*	TGGTGATGCATAGGTCGTTGACCA	AGGGAGAATTTGGATGGGTGGGTT
AT4G39070	*BZS1*	GATCGCCATGTTCATTTCGC	TAATAATGCACGCCTCTCCC
AT2G46970	*PIL1*	AAATTGCTCTCAGCCATTCGTGG	TTCTAAGTTTGAGGCGGACGCAG
AT4G16780	*ATHB-2*	GTCGTTGCCGGTCAATGC	CCTAGGACGAAGAGCGTCAAAA
AT4G14130	*XTR7*	CACCGTCACTGCTTACTACTTG	CATTGGTGTGAAGAACATAAG
AT4G32280	*IAA29*	CACCATCATTGCCCGTATCA	CCACAGTAGCCGTTGTTGGA
AtCG00680	*psbB*	GAATTAGATCGTGCGACTTTGA	CTAGCACCATGCCAAATGTGTC
AtCG00490	*rbcL*	GGAGATGATTCTGTACTACAAT	GTCCCTCATTACGAGCTTGTAC
AtCG00190	*rpoB*	CTAGTGGACATTATGCACTTGT	CAGATTTATAAGTAAGCATCTCTTG
AtCG00180	*rpoC1*	GATGCAATTGGAGCTTATCG	CGATAGGAACTTCTCTTGAAGC

Supplementary Table 3. PCR primers used for making the GST pulldown constructs.

Accession	Gene name	Vector	Forward primer	Reverse primer
AT2G34640	*HMR*	pET42b	GGCGAATTCTATGGCGTCAATATCAACCACCAC	GGCCTGCAGTTAAGGATCAGTCTCCTCTTCAAAG
AT2G34640	*HMRΔ115*	pET42b	GGCGAATTCTCCTGAAGGAACTTCGAGCCG	GGCCTGCAGTTAAGGATCAGTCTCCTCTTCAAAG
AT2G34640	*HMRΔ251*	pET42b	GGCGAATTCTATGCCTGATGTTGAAACGGTTTTCCTC	GGCCTGCAGTTAAGGATCAGTCTCCTCTTCAAAG
AT4G28590	*RCB*	pCMX-PL2-NterHA	TCTCGAGAAGCTTGATATCGCTATGAGTTTCTTCGCT GTTGCTTG	GCTACTAGCTAGCTGGCCAGCTAACAGTACGGG GTTACATTAG
AT4G28590	*RCBΔ51*	pCMX-PL2-NterHA	TCTCGAGAAGCTTGATATCGCTTCTGATTCCGTACTA GACCCTG	GCTACTAGCTAGCTGGCCAGCTAACAGTACGGG GTTACATTAG
AT4G28590	*RCBΔ89*	pCMX-PL2-NterHA	TCTCGAGAAGCTTGATATCGCTGTCCCGCGTAAATC GAAGC	GCTACTAGCTAGCTGGCCAGCTAACAGTACGGG GTTACATTAG
AT4G28590	*RCBΔ158*	pCMX-PL2-NterHA	TCTCGAGAAGCTTGATATCGCTCCAGATTGGCCAGTA GATACAG	GCTACTAGCTAGCTGGCCAGCTAACAGTACGGG GTTACATTAG
